# Peculiarities of the Third Natural Frequency Vibrations of a Cantilever for the Improvement of Energy Harvesting

**DOI:** 10.3390/s150612594

**Published:** 2015-05-28

**Authors:** Vytautas Ostasevicius, Giedrius Janusas, Ieva Milasauskaite, Mindaugas Zilys, Laura Kizauskiene

**Affiliations:** 1 Institute of Mechatronics, Kaunas University of Technology, Studentu 56-123, Kaunas LT-51368, Lithuania; E-Mails: vytautas.ostasevicius@ktu.lt (V.O.); ieva.milasauskaite@gmail.com (I.M.); 2 Faculty of Mechanical Engineering and Design, Kaunas University of Technology, Studentu 56-338, Kaunas LT-51368, Lithuania; 3 Faculty of Electrical and Electronics Engineering, Kaunas University of Technology, Studentu 48-211, Kaunas LT-51368, Lithuania; E-Mail: mindaugas.zilys@ktu.lt; 4 Faculty of Informatics, Kaunas University of Technology, Studentu 48-213, Kaunas LT-51368, Lithuania; E-Mail: laura.kizauskiene@ktu.lt

**Keywords:** vibro-impact motion, optimal configuration, vibration energy harvesting, piezoelectric layer, resonant frequency, laser vibrometry, holography system

## Abstract

This paper focuses on several aspects extending the dynamical efficiency of a cantilever beam vibrating in the third mode. A few ways of producing this mode stimulation, namely vibro-impact or forced excitation, as well as its application for energy harvesting devices are proposed. The paper presents numerical and experimental analyses of novel structural dynamics effects along with an optimal configuration of the cantilever beam. The peculiarities of a cantilever beam vibrating in the third mode are related to the significant increase of the level of deformations capable of extracting significant additional amounts of energy compared to the conventional harvester vibrating in the first mode. Two types of a piezoelectric vibrating energy harvester (PVEH) prototype are analysed in this paper: the first one without electrode segmentation, while the second is segmented using electrode segmentation at the strain nodes of the third vibration mode to achieve effective operation at the third resonant frequency. The results of this research revealed that the voltage generated by any segment of the segmented PVEH prototype excited at the third resonant frequency demonstrated a 3.4–4.8-fold increase in comparison with the non-segmented prototype. Simultaneously, the efficiency of the energy harvester prototype also increased at lower resonant frequencies from 16% to 90%. The insights presented in the paper may serve for the development and fabrication of advanced piezoelectric energy harvesters which would be able to generate a considerably increased amount of electrical energy independently of the frequency of kinematical excitation.

## 1. Introduction

A literature review reveals that cantilevers vibrating in higher modes have so far received only moderate attention. Nevertheless, the dynamic response of a cantilever-type vibro-impacting system, such as speed of operation, stability, reliability, longevity, *etc*. is an extremely important topic amongst the other ongoing research topics in the field of energy harvesting, because designing a commercially viable device is possible only by means of comprehensive awareness and precise prediction of its dynamic aspects [1]. Special attention should be paid to the peculiarities of optimal structures excited by the wider frequency diapason caused by impacts. The dynamic characteristics of the vibro-impacting system are often influenced by the system parameters, thus in [2] the authors emphasize and analyse the need for a theoretical model of the cantilever beam vibro-impacting system. They employ numerical methods to determine the influence of clearance, damping and nonlinear items on the dynamic characteristics of the vibro-impacting system and the results have practical applications, exploiting the nonlinear phenomena and the instability mechanism of the systems. Further exploring the research in the field of vibro-impacting system dynamics, one may come across papers proposing a methodology for an automatic choice of the measurement locations of a nonlinear structure that needs to be monitored while operating [3], deriving piecewise-linear models with a single degree of freedom for a driven vertical cantilever beam with a localized mass and symmetric stops [4], introducing modelling framework that is suitable for resolving the singularities of impact encountered in practical applications [5], or developing novel optimized cantilever beams with enhanced travel ranges [6,7]. 

Rapidly increasing numbers of wireless sensor nodes and their networks (WSNs), ranging in applications of structural health (e.g., cracks in buildings), industrial environments (e.g., machining processes), household/weather conditions (e.g., temperature and humidity) and even human health and well-being monitoring have kept energy harvesting as a target of researchers for many years [8,9,10]. 

The researchers in [11] aim to propose inexhaustible energy harvesters efficient enough to power wireless sensor nodes (the power ranges of which may reach hundreds of microwatts in active operation modes), which would address what is currently the most expensive issue of WSN node maintenance, *i.e*., battery replacement. The latter issue becomes more prominent with increasing numbers of wireless devices causing environmental hazards due to battery chemical waste. It goes without saying that while one might easily consider changing the battery of a temperature sensor located on a windowsill, yet the very same action may become fatal when one considers battery replacement in heart pacemakers or other implantable devices. Moreover, an abrupt failure of such a WSN node due to power run-out could lead to critical incidents. Thus, self-powered systems would not only be beneficial from the financial point of view, but would address human and environmental safety issues as well.

A variety of ambient energy sources—light, wind and hydropower, thermal gradients and kinetic energy—have been probed for energy harvesting applications [12,13]. The choice of the most appropriate, reliable and efficient energy source relies mainly on the foreseen sensor node utilization, e.g., solar energy may efficiently power weather monitoring sensors in bright outdoor areas, but it would be useless in a dark and dirty factory shop-floor. As the investigated energy harvester could be utilized in machining process monitoring, kinetic energy was chosen as the driving energy source. Piezoelectric transduction was selected from other available (electrostatic and electromagnetic) motion-to-electricity conversion methods due to its high energy density, favourable dynamic response, self-contained power generation (electrostatic generators would require an initial bias voltage to operate) and relative ease of integration as well as production employing microelectromechanical system technologies. Despite the intense theoretical and experimental research, piezoelectric energy harvesters are still not widely used in practical applications since they often suffer from inefficiency and unreliability in real environment applications. Thus, various techniques and methods are used to improve the efficiency of piezoelectric energy harvesters. 

The results presented in this paper fall into the group of research on optimal structural design of harvesters as it concerns a structural modification of harvester design via electrode segmentation of a piezoelectric vibrating energy harvester (PVEH) in order to boost its output voltage. The paper combines the authors’ knowledge on coupled-field nonlinear contact-type piezoelectric transducer modelling and simulation [14], experimental studies of coupled dynamic and electric aspects of the PVEH under variable resistive load [11] as well as hybrid numerical-experimental optical techniques, which are applied for the characterization of nonlinearity in micro-cantilevers [15], presented in previous papers. Moreover, the research covers some aspects of classical vibration theory which suggests that higher vibration modes of the cantilever beam have strain nodes, where the dynamic strain distribution changes sign. It is assumed that if these strain nodes are covered by continuous electrodes, cancellation of electric outputs occurs, resulting in an overall reduction of the harvested energy. This issue does not arise if a piezoelectric energy harvester is operating mainly at its first resonant frequency (strain distribution function in piezoelectric layers would not change the sign in the first vibration mode); however, the problem becomes more prominent with higher vibration modes. 

As has been mentioned, this paper focuses on the dynamic aspects of constant cross-section and optimally shaped cantilever beam vibro-impacting structures intended for higher operational speed devices or energy harvesting applications, and on the design and production of a prototype of the piezoelectric energy harvester with segmented electrodes for optimal operation at the third resonant frequency.

## 2. Design of the Energy Harvester Prototype

**Figure 1:**
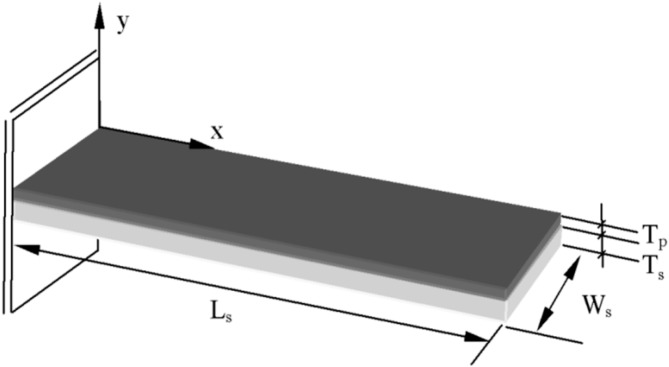
Scheme of the modelled energy harvester prototype.

To show the possibility of effectively using a cantilever vibrating in the third mode for energy harvesting, its Finite Element model was developed with the Comsol Multiphysics software. Simulations were used to determine resonant frequencies, natural vibration modes as well as preliminary location of the strain nodes of the vibrating structure. Figure 1 presents a scheme of the FE model which is based on a stainless steel rectangular cantilever beam (*L_s_* = 37 mm, *T_s_* = 0.36 mm, *W_s_* = 11.05 mm) with one T107-H4E-602 piezoelectric layer bonded on its top surface (*L_p_* = 37 mm, *T_p_* = 0.191 mm, *W_p_* = 10 mm). 

**Figure 2:**
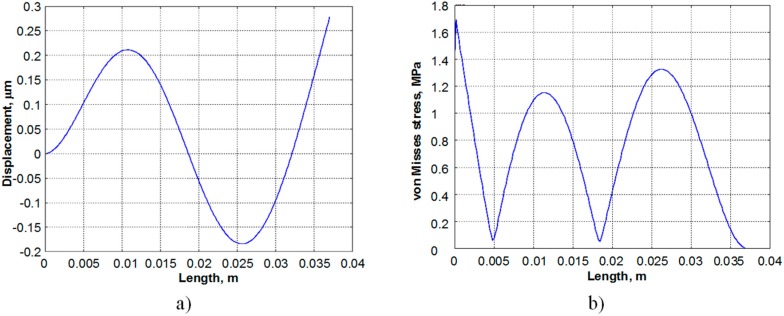
Deformed shape (**a**) and von Misses stress distribution (**b**) for the third vibration mode of the energy harvester prototype.

Simulation results are presented in Figure 2. It shows the deformed shape and von Misses stress distribution in the third vibration mode of the PVEH prototype. As has been mentioned in the introductory part, charge cancelation in piezoelectric materials does not appear in the first vibration mode (strain distribution function does not change the sign when the harvester is operating at its first natural frequency), thus higher vibration in the third vibration mode (Figure 2a). As the von Misses stress distribution plot in Figure 2b suggests, the simulated locations of the strain nodes in the third vibration mode are 6 mm and 19 mm from the clamping point, respectively.

To summarize, the energy harvester prototypes produced for the experimental purposes are characterised by: (a) non–segmented electrodes attached to the piezoelectric layer; (b) electrodes segmented at 6 mm and 19 mm from the clamping point, corresponding to the strain nodes of the third vibration mode.

### 2.1. Research Objects and Experimental Setup

**Figure 3:**
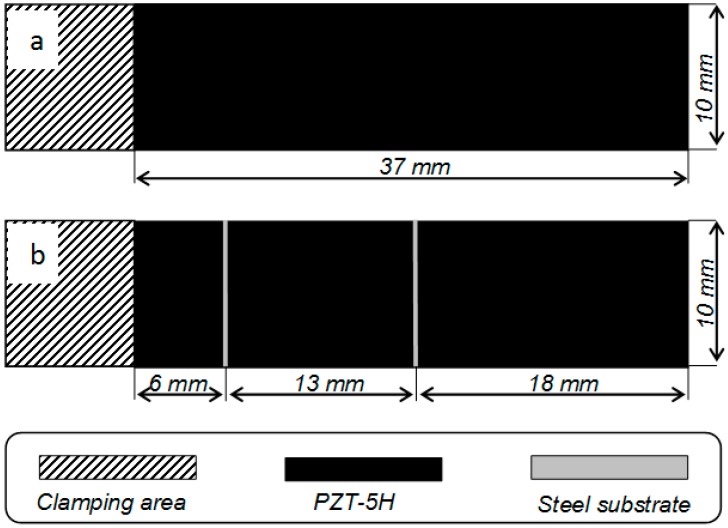
Schemes of elaborated piezoelectric energy harvester prototypes: (**a**) non-segmented; (**b**) segmented for operation in the third vibration mode.

Figure 3 presents drawings of the PEH prototypes built for the experimental study. Both prototypes were built from T107-H4E-602 plate (Piezo Systems, Inc., Woburn, MA, USA) covered with conductive layers. The plate was cut to the appropriate dimensions with an automatic scriber and glued to a stainless steel substrate. The first harvester prototype (Figure 3a) had no electrode segmentation, while the second PVEH prototype (Figure 3b) featured electrode segmentation configured for a device operating at the third resonant frequency. The electrode of the piezoelectric material was divided into three parts at the strain nodes of the third vibration mode in order to boost its output voltage. If these strain nodes were covered by continuous electrodes, cancellation of electric outputs occurred, resulting in an overall reduction of the harvested energy.

**Figure 4:**
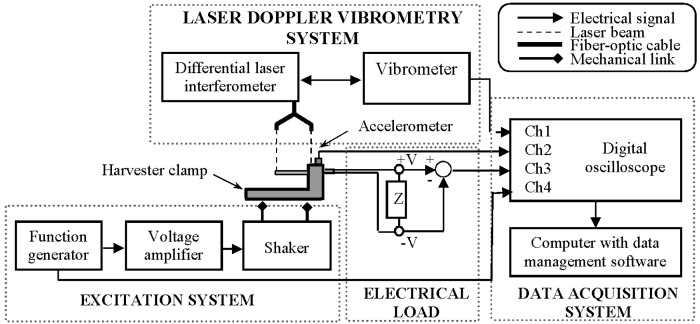
Scheme of the laser Doppler vibrometry system.

**Figure 5:**
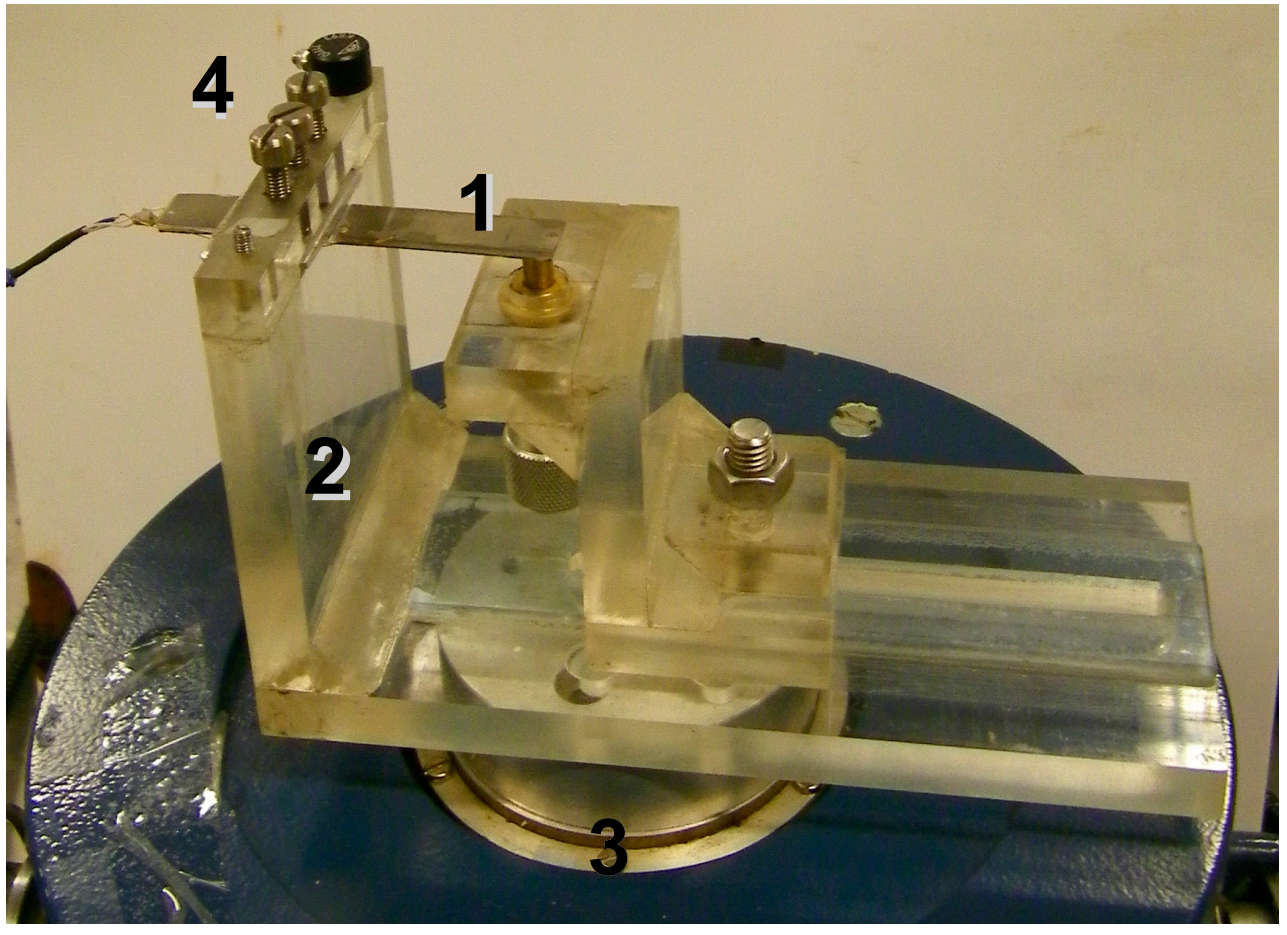
Experimental setup for the analysis of the vibration energy harvester prototype response to harmonic excitation: 1–vibration energy harvester prototype, 2–harvester clamp, 3–electromagnetic shaker, 4–accelerometer.

Tip velocity (or displacement) of the fabricated prototype in the transverse direction was experimentally analysed using Doppler vibrometry (Figure 4). The measurement system consisted of the PVEH prototype fixed in a custom-built clamp (Figure 5) and three main subsystems–excitation system, measurement system and data gathering system. The PVEH prototype was excited by an electromagnetic shaker, while its excitation was controlled by a 33220A function generator (Agilent, Santa Clara, CA, USA) and a VPA2100MN voltage amplifier (HQ Power, Gavere, Belgium). A single-axis KS-93 piezoelectric accelerometer (Metra, Radebeul, Germany; sensitivity–0.35 mV/(m/s^2^)), which was fixed on the top of the clamp, was used for measurement of the prototype excitation. Tip velocity (or displacement) of the prototype was measured by an OFV-512 differential laser interferometer (Polytec, Waldbronn, Germany) connected to a Polytec OFV-5000 vibrometer controller. All data were gathered by a 3425 USB oscilloscope (Pico, St Neots, UK) and pre-processed as well as visualized by the PicoScope Oscilloscope Software 6.10.11.

### 2.2. Results

**Figure 6:**
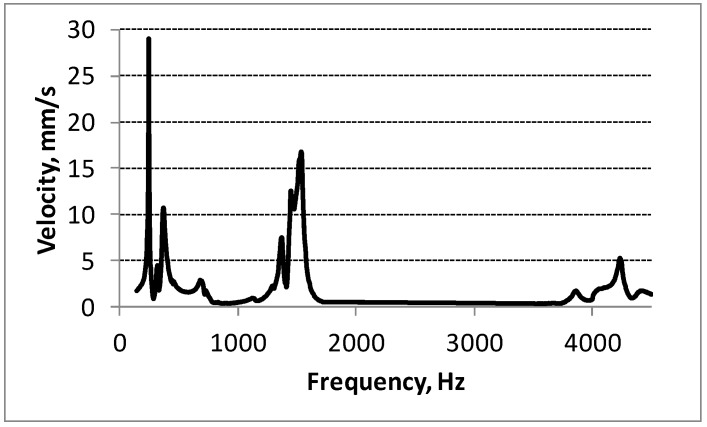
Measured frequency response of tip velocity of the PVEH prototype.

Figure 6 shows the measured frequency response of tip velocity of the PVEH (the energy harvester was excited by a 3 g force). It was found that the first resonant frequency of the harvester is 252 Hz, the second is 1452 Hz and the third one—4231 Hz. 

Some minor peaks or minimums have been noticed in each resonant frequency range due to the boundary conditions of clamping and beating phenomenon when excitation frequency is near resonant frequency of energy harvester. The same experimental conditions were used when registering the voltages generated by the non-segmented energy harvester.

**Figure 7:**
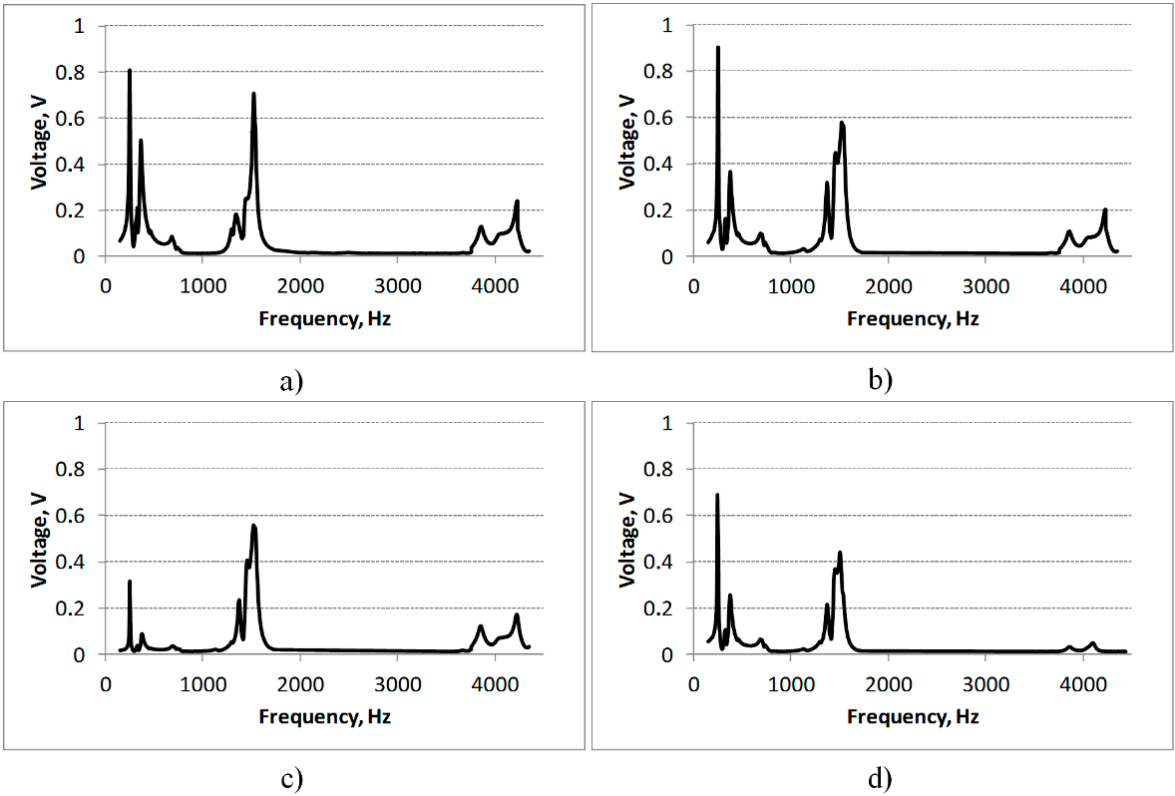
Measured frequency response of output voltage of the first (16 mm, **a**), second (13 mm, **b**) and third (18 mm, **c**) segment generated by the segmented energy harvester in comparison with the frequency response of output voltage of the non-segmented energy harvester (**d**).

The measured output electrical potential (electrical load resistance of 4700 Ω, arbitrarily selected) of the non-segmented energy harvester (Figure 7d) at the first resonant frequency was 0.69 V, at the second resonant frequency—0.37 V and at the third—0.05 V. These results show that the best operating frequencies for a non-segmented energy harvester are 252 Hz and 1452 Hz, while at higher frequencies this harvester is useless.

Therefore, according to the numerical results described in Section 2, the segmented energy harvester was elaborated (Figure 3b). It has three electrodes: 6 mm, 13 mm and 18 mm length that are located between the strain nodes of the harvester vibrating at the third resonant mode. The same experiments (excited by a 3 g force, electrical load resistance of 4700 Ω) were performed with the segmented energy harvester. Thus, Figure 7 provides the measured frequency responses of output electrical potential of the first (6 mm), the second (13 mm) and the third segment (18 mm) of the segmented energy harvester.

The graphs show that voltage, generated by any segment of the segmented energy harvester excited at the third resonant frequency (Figure 7a–c), increased from 3.4 (0.17 V for the third segment) to 4.8 (0.24 V for the first segment) times in comparison with the non-segmented one. Simultaneously, the efficiency of the energy harvester also increased at lower resonant frequencies. The voltage generated by the first segment rose by 14% in comparison with the non-segmented PVEH and by 57% at the second resonant frequency. As follows, the voltage generated by the second segment increased by 28% and by 33% at the second resonant frequency. In fact, only the voltage generated by the third segment fell by 55% in comparison with the non-segmented PVEH and rose by 27% at the second resonant frequency.

If the voltages generated by separate segments could be added electrically, then the segmented energy harvester would generate 2.94 times higher voltage than the non-segmented one at the first resonant frequency, 4.2 times at the second and 12.2 times at the third. These experiments show that a greater area of the piezoelectric layer on the energy harvester does not necessarily result in higher generated voltages, *i.e.*, optimal location and size of the piezoelectric layer on the substrate should also be defined when designing a piezoelectric energy harvester. 

## 3. Experimental Setup for Vibro-Impact

**Figure 8:**
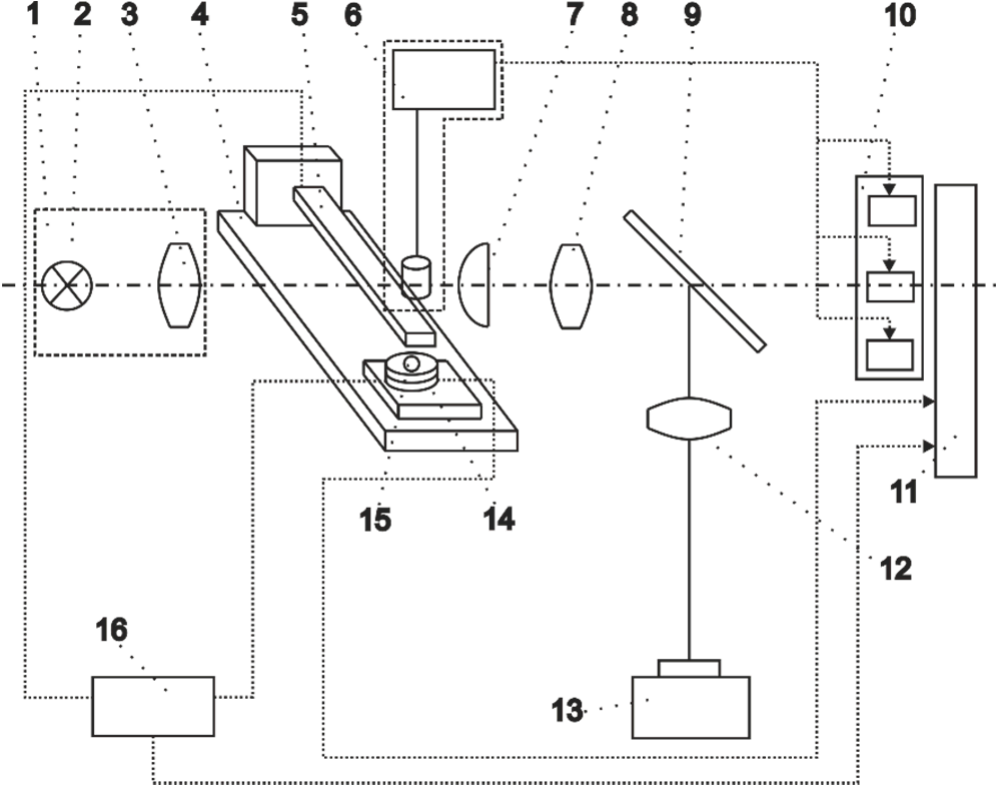
Vibro-impact experimental setup.

The specially designed and custom-built experimental setup for vibro-impact investigation of a steel cantilever is featured in Figure 8. The cantilever specimen is formed of a rectangular stainless steel bar (*E* = 209 GPa, *ν* = 0.3, *ρ* = 7917 kg/m^3^) *H*x*B* = (0.5 × 8.8) × 10^−3^ m and length *L* = 80.0 × 10^−3^ m.

The light reflector 1 is composed of a light source 2 and condensing lens 3; the vibro-impact system consists of a cantilever 5 and support 15. The guideways of the frame 4 enable one to change the position of the cantilever in the plane of transverse vibrations in two directions perpendicular to each other. The cantilever is shifted by using a mechanical microscrew that enables accurate estimation of the distance between the contact surfaces of the cantilever and the support. The light source 2 exposes the cantilever 5, which is actuated from a statically deflected position by the triggering block 6. A shadow image of the cantilever 5 is enlarged in a transverse direction by the cylindrical lens 7 and 8, then it is projected on the input surface of the photoelectric transducer of displacement 10 by means of the light-refracting screen 9 and the objective 12 on the photo recorder 13.

The working principle of the device is as follows: the triggering attachment 6 actuates the cantilever 5 and runs a block of displacement photoelectric transducers 10. As the cantilever moves, its several times magnified shadow changes the input light intensity on the displacement transducer 10 and later generates the output of alternating signal, corresponding to the laws of motion of the characteristic points of the vibrating cantilever. At the same time, the triggering attachment 6 runs the photo recorder 13 and the modes of vibration of the cantilever are registered on the film.

At the moment when the cantilever 5 bounces the support 15, the impact sensor 14 measures the contact pressure and transmits the signals to the recorder 11. At the same time, the gauge 16 measures the dynamic resistance between the cantilever 5 and the support 15.

**Figure 9:**
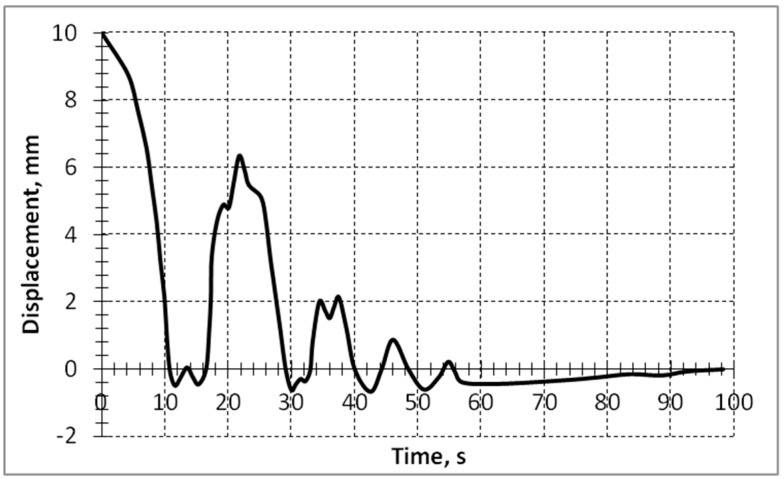
Experimental trajectory of the vibro-impact motion of the cantilever.

Figure 9 features the motion trajectory of the cantilever 5 in case of free impact vibrations when upon the release from the deformed position (initial deflection 10 mm), the cantilever makes an impact against the rigid immovable support 15. 

**Figure 10:**
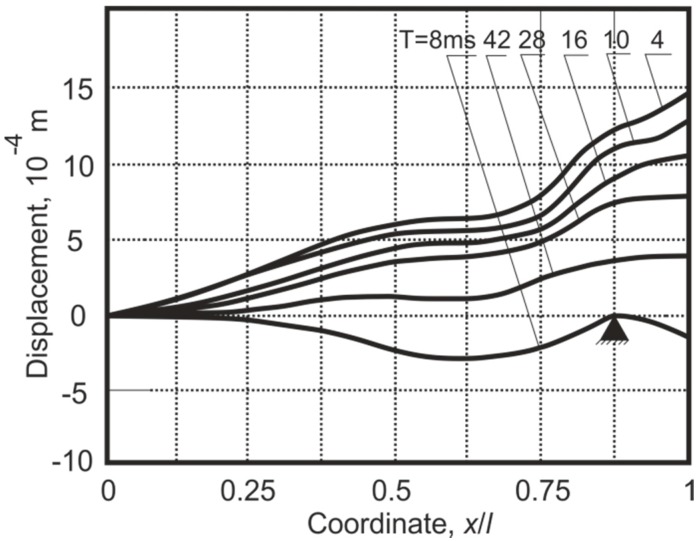
Dependence of the cantilever rebound amplitudes *y_max_* on the support position *x*/*l* at fixed time moments *T* after rebound.

The position of the immovable support could be changed by shifting it in the direction of the cantilever axis. By placing the immovable support in certain fixed positions, related to the nodal point positions of the higher vibration modes of the cantilever, the amplitudes of these vibration modes can be markedly increased. Figure 10 presents the cantilever vibration momentum *T* curves after a collision with the support in the third mode nodal point *x*/*l* = 0.87, where *x* is the distance of the support from the fixed end of the cantilever and *l* is the length of the cantilever. 

**Figure 11:**
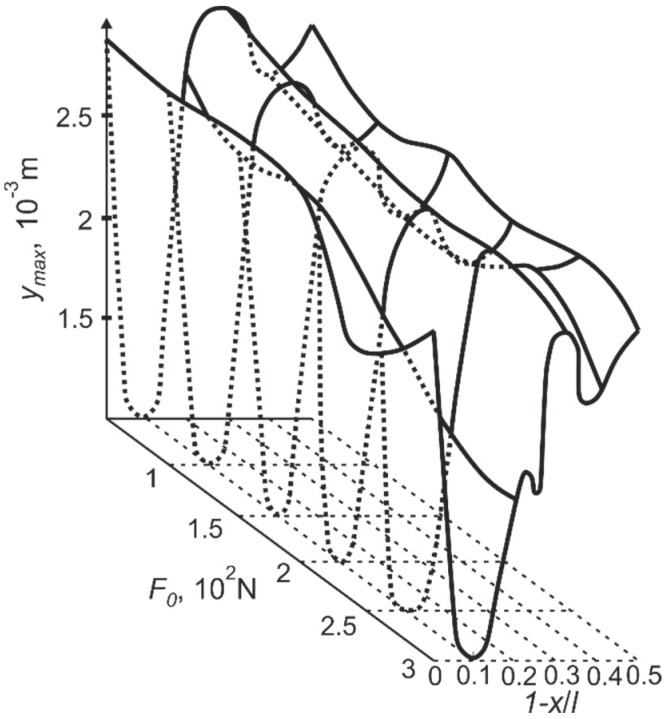
Dependence of the cantilever rebound amplitude *y_max_* on the position of the support 1-*x*/*l* (distance of the support from the free end of the cantilever) and the pre–stress of the cantilever *F_0_* on the support.

This means that the location of the support in the nodal point of the third vibration mode is beneficial for such a vibro-impact system, because these dominating flexural cantilever vibrations are similar to the cantilever vibration at the third natural frequency. The domination of the third mode does not mean that the first two modes are cancelled, but rather they are suppressed. A considerable increase of the third mode amplitudes results in the intensification of the internal energy dissipation in the structure material. This could be confirmed by the drastically decreased cantilever rebound amplitudes when the support was located in the nodal point of the third mode at *x*/*l* = 0.87 (the gap between the deformed position of the cantilever and the support *q* = 10^−4^ m) (Figure 11). 

**Figure 12:**
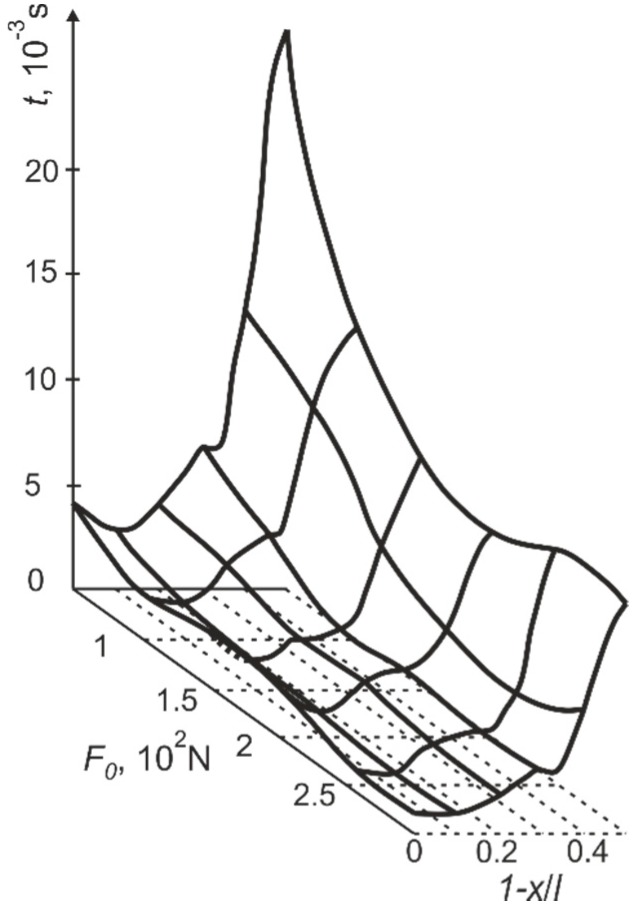
Dependence of vibro-impact motion time on the position of the support 1-*x*/*l* (distance of the support from the free end of the cantilever) and the pre-stress *F_0_* of the cantilever on the support.

The presented explanation is also confirmed by the dependence of vibro-impact motion time on the position of the support *x*/*l* and the pre-stress *F_0_* of the cantilever on the support (the gap between the deformed position of the cantilever and the support *q* = 10^−4^ m) (Figure 12). As the amplitudes of the cantilever rebounds fall when the support is located at the nodal point of the third vibration mode, the time of vibro-impact motion or transitory period decreases too.

**Figure 13:**
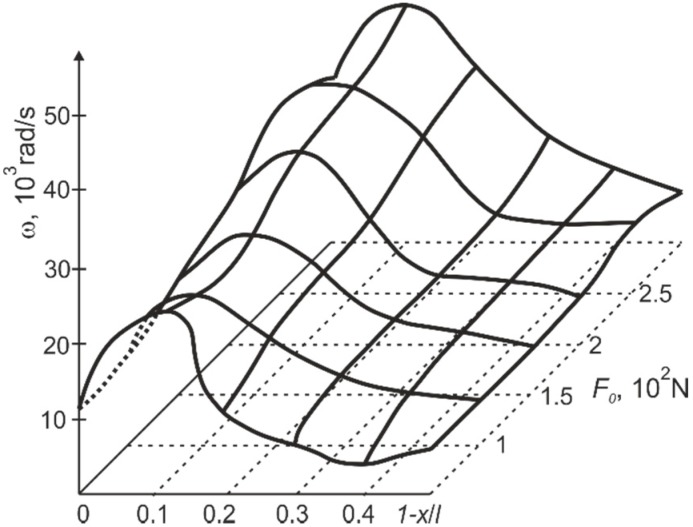
Dependence of free impact vibration frequency of the cantilever on the position of the support 1-*x*/*l* (distance of the support from the free end of the cantilever) and the pre–stress F0 of the cantilever on the support.

The position of the support has a marked effect on the frequency of the free impact vibrations of the cantilever. Figure 13 suggests that vibration at maximum frequency is characteristic if the support is positioned in the point *x*/*l* = 0.87, *i.e.*, in the nodal point of the third vibration mode (the gap between the deformed position of the cantilever and the support *q* = 10^−4^ m). The frequencies of free impact vibrations also change at different pre-stress *F_0_*. 

In the case when there is no pre-stress, the frequency of free impact vibration of the cantilever is equal to the first free transverse vibration frequency. 

## 4. Cantilever Optimization

Sometimes there is a need to create a structure whose natural frequencies would fit in a certain interval (*ω_min_*; *ω_max_*). This can be due to many reasons, for example, a vibrating piezoelectric energy harvester generates higher power output values while it is operating at its resonant frequency, coinciding with the ambient source frequency. 

**Figure 14:**
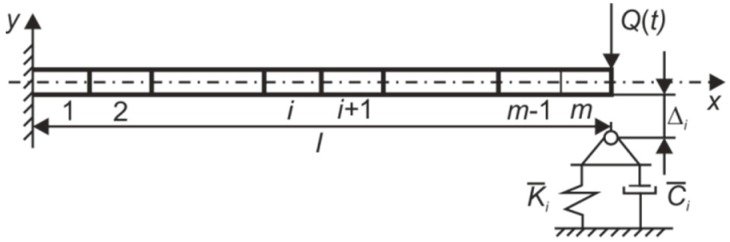
Scheme of the developed 2-D finite element model of the impacting cantilever.

Figure 14 represents a 2-D computational scheme of the cantilever with the support. The model is made from *m* linear beam elements located in a single layer. Each finite element has three degrees of freedom at each node. External air pressure forces are neglected in the model, therefore *Q*(*t*) is only a mechanical load which acts in the system. ${\bar{K}}_{i}$ and ${\bar{C}}_{i}$ are stiffness and viscous damping coefficients of the support, $\Delta_{i}$*-*size of the gap between the *i-*th nodal point of the structure and the surface of the support located at the *i*-th nodal point. 

The target function in such a case is as follows:

(1) $$\text{Φ }\left(\text{A}\right)=\min\text{ρa }\left({\text{A}}_{1}+{\text{A}}_{2}+\;\ldots +{\text{ A}}_{\text{m}}\right)$$

where *A_i_* — cross-sections of structure components. When the method of nonlinear programming, e.g., gradient projection, is used, the unequally-shaped constraints are employed:

(2) $$f_{m}\left(A\right)=1-A_{m}\;/\;A_{i\;}\leq 0$$

(3) $$f_{m+1}\left(\omega \right)=1-\omega \;/\;\omega^{\text{*}}\leq 0$$

where ω*—the prescribed frequency of structure vibrations. 

**Figure 15:**
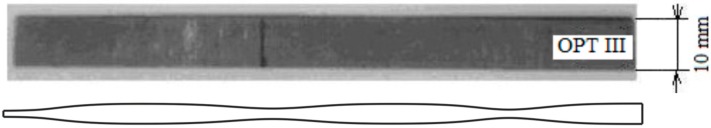
Technical drawing of the fabricated cantilever.

The optimal configuration of the cantilever was achieved using the method of gradient projection. Figure 15 provides the optimal structure obtained for the defined frequency of the third (*ω*_3_) natural transverse vibration.

From the sketch in Figure 15 the distances from the minimum and maximum cross-sections to the fixing site of the structure are easy to measure. The minimum cross-section of the structure that is optimal at the third value of transverse vibration frequency *ω*_3_ is located the distances of 0.15*l* and 0.5*l* from the fixed (right) end of cantilever, where *l* is its length.

For the purposes of this research, an optimally configured cantilever (Figure 15) with an optimized cross-section for the operation at the third resonant frequency was fabricated and analysed. The optimal cantilever configuration was obtained to solve the optimization problem as per the above described theory with the aim of mass minimization (yet the diminution of the cantilever was limited) in the presence of a constraining equation system. The state of the system is described by a mode analysis equation, demanding that the natural frequencies of the second and the third flexural vibration would coincide with the corresponding frequency values of the constant cross-section cantilever. 

The cantilevers were fabricated employing a WJ 3030-2Z water jet cutting system (PTV, Hostivice, Czech Republic), because it allows cutting steel into intricate shapes without exposing the work piece to heat during cutting, thus no tension is created in the cut area.

## 5. Frequency Response Analysis 

**Figure 16:**
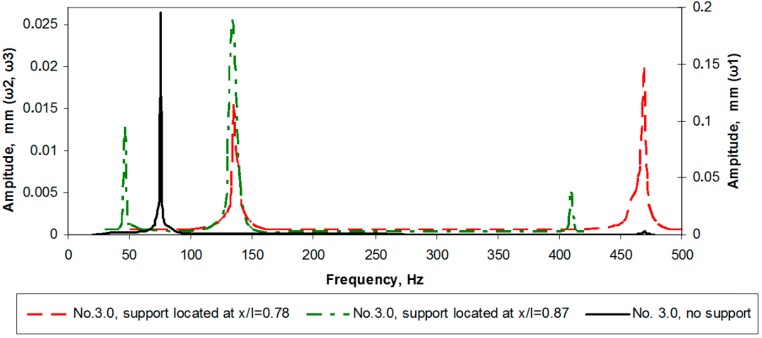
Amplitude-frequency characteristics for the constant cross-section cantilever.

For the disclosure of the peculiarities of the optimally configured cantilever, first of all, it was necessary to test the dynamics of constant cross-section cantilever (Figure 16). The first natural mode of the unsupported constant cross-section cantilever was registered at 75 Hz, the second mode at 470 Hz, while the third mode was not fixed at all. It goes without saying that the first resonance vibration amplitudes of the unsupported cantilever were significantly greater (up to 10 times) compared to the supported ones. As the support was located at *x*/*l* = 0.78, the second amplitude peak was noted at 469 Hz (vibration amplitude is 10 times higher as compared to the unsupported cantilever). When the support was shifted to *x*/*l* = 0.87, the first resonance was noted at 46 Hz and the second one at 410 Hz (with both vibration amplitudes smaller as compared to the support located at *x*/*l* = 0.78). For the latter two cases with the support, the amplitude peak was noted at ~135 Hz; however, this is the natural frequency of a cantilever clamp, which was excluded from further research.

**Figure 17:**
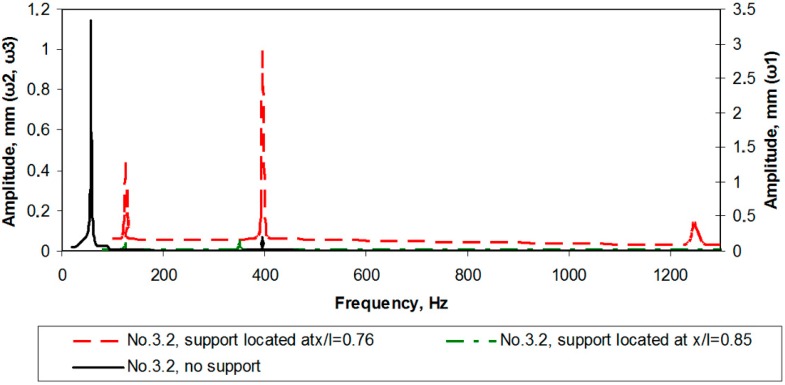
Amplitude-frequency response characteristics for the optimally configured cantilever without the support and with support located at *x*/*l*~0.78 and *x*/*l*~0.87.

Next, amplitude-frequency characteristics for the optimally configured cantilever (Figure 17) were obtained. For the unsupported cantilever the first resonant frequency was registered at 58 Hz, the second at 396 Hz and the third one at 1336 Hz (the vibration amplitude of the first resonance was the highest for the unsupported cantilever). In case of the support introduced at *x*/*l* = 0.78, the second resonance was noted at 395 Hz and the third at 1249 Hz (the amplitude of the second resonance was the highest). As the support was shifted to *x*/*l* = 0.87, the second resonance was registered at 350 Hz, the third at 1325 Hz. A natural cantilever clamp resonance was registered again (at ~126 Hz) and excluded from further investigation.

## 6. Cantilever Resonant Frequency Determination and Oscillation Analysis by Means of PRISM Holography System

**Figure 18:**
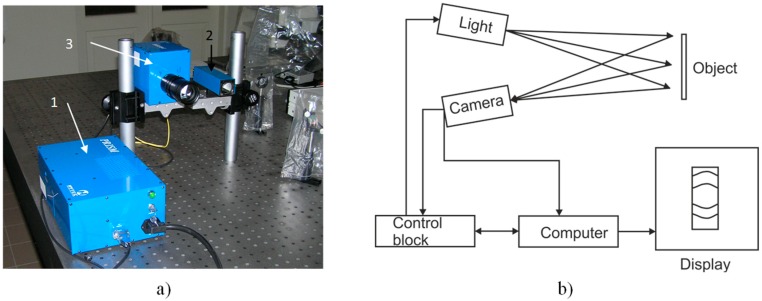
Holography System PRISM: (**a**) set-up picture; (**b**) operation scheme.

A Precise Real-Time Instrument for Surface Measurement (PRISM) holography system (Hytec, Los Alamos, NM, USA), presented in Figure 18, was used for the determination and oscillation analysis of the cantilever resonant frequency. The PRISM allows completing and processing experimental measurements in less than 5 min as well as is capable of determining displacements of less than 20 nm [16]. 

The main characteristics of the PRISM system are presented in Table 1.

**Table 1:** Main characteristics of the PRISM system.

Measurement Sensitivity	<20 nm
Dynamic measurement boundary	100 µm
Measurement boundary	>100 µm
Greatest measurement area	1 m diameter
Distance to the object	>¼ m
Data registration frequency	30 Hz

The main part of the system is a control block, which splits green (532 nm) semiconductor laser beam into two beams—object and reference. Lenses are used to control the object beam and light falling on the object. Light, reflected back from the object, is registered by a camera, which combines object and reference beams, registering the interference pattern (ratio of the object and reference beams may be altered in order to achieve the best definition of interference bands). Interference pattern is transferred to the computer, where it is processed with the PRISMA-DAQ software, allowing one to monitor real time dynamic processes occurring at the research object as well as deformations caused by the internal and external forces. For this experiment, an acoustic field was employed to excite the cantilever by ranging the harmonic excitation signal from 10 Hz to 2000 Hz. The shapes and amplitudes of the cantilever surface deformation were determined by analysing the interference patterns.

**Figure 19:**
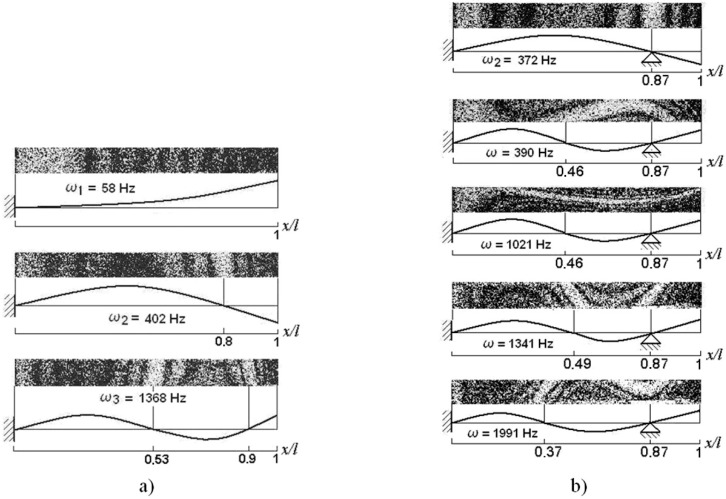
Optimally configured cantilever: natural vibration modes and frequencies: (**a**) unsupported; (**b**) rigid support at (*x*/*l* = 0.87); *ω*_1_–I mode; *ω*_2_–II mode; *ω*_3_–III mode.

The set of experiments was performed with the optimally configured cantilever, whose holograms are presented in Figure 19. For this configuration the unsupported cantilever was excited at its first resonant frequency at *ω*_1_ = 58 Hz, the second resonance at ω_2_ = 402 Hz and the third one at *ω*_3_ =1368 Hz. In case of the support located at *x*/*l*~0.78, the second vibration mode was excited at *ω*_2_ = 372 Hz. Further increasing vibration frequency, the next resonances were noted at *ω*= 390, 1021, 1341 and 1992 Hz (the cantilever was flexing and twisting). It should be noted that *ω* = 1341 Hz excited the cantilever torsion to one direction, while at *ω* = 1991 Hz the cantilever was already turning to the other direction. It may be assumed that the third cantilever resonance occurs at 1341 Hz frequency, while *ω* = 1991 Hz excites higher cantilever vibration mode.

## 7. Comparison of Doppler Vibrometry and Holography Experimental Results

Table 2 compares natural frequencies of constant cross-section and optimal configuration of the cantilever measured with the Doppler vibrometry system and natural frequencies obtained with the PRISM holography system. As may be seen from the data presented in Table 2, the values of natural frequencies are in a close agreement (2%–3% value fluctuation), thus a conclusion can be made that the performed measurements are qualitative.

**Table 2:** Summary of natural frequencies for different cantilever configurations measured with the Doppler vibrometry system and the PRISM holography system.

Cantilever Configurations	Frequency	Natural Frequencies, Hz				
		Measured with Doppler Vibrometry System			Measured with PRISM Holography System	
		Support Location			Support Location	
		Unsupported	*x*/*l* ≈ 0.78	*x*/*l* ≈ 0.87	Unsupported	*x*/*l* ≈ 0.87
Constant cross section	ω_1_	75	-	46	77	-
	ω_2_	470	469	410	480	465
	ω_3_	-	-	-	1335	1186
Optimal	ω_1_	58	-	-	58	-
	ω_2_	396	395	350	402	372
	ω_3_	1336	1249	1325	1368	1341, 1991

**Figure 20:**
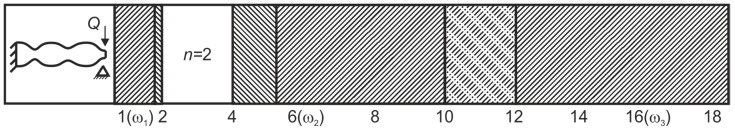
Periodically excited vibro-impact motion laws of the optimal cantilever.

Another quality of the presented technique important for the experimentation is the possibility to identify diverse vibro-impact lows of the optimally configured cantilever and to show the possible ways of increasing its harvesting capabilities. A sketch of such a configuration is presented on the left side of Figure 20 for the prescribed frequency of the third natural transverse vibrations.

Under the sine excitation Q, the optimally shaped cantilever hits the support which is placed at its free end and starts the vibro-impact motion. On the right side of Figure 20, the particularly settled areas are presented at different excitation frequencies *ω*/*ω*_1._ When excitation frequency ω is equal to the first natural frequency ω_1_ of the cantilever, the first resonance of such a vibro-impact system has a place and the area of the settled periodic vibro-impact motion (right-hand dashes) appears in the narrow diapason of the excitation frequencies. When the excitation frequency increases, the vibro-impact motion becomes close to the settled one (points and dashes), with its areas alternating with unsettled or chaotic recurrent vibration laws (left-hand dashes). In between of these two chaotic regimes, the *n* = 2 non-dashed wide area is characterized by the settled vibro-impact motion, when in the course of two sine excitation periods, the optimal cantilever makes only one impact on the support. When the excitation frequency coincides with the second natural frequency of the cantilever, the second resonance is characterised by the settled vibro-impact motion. However, the most beneficial is the third resonance characterised by a very large diapason of the settled vibro-impact motion. This phenomenon could be useful for increasing energy harvesting efficiency, as well as for creating high speed vibro-impact mechanisms.

## 8. Conclusions

This paper is focused on the vibro-impacting of constant cross-section and optimally shaped cantilever as well as on the design and production of a piezoelectric energy harvester prototype with segmented electrodes for its optimal operation at the third resonant frequency. Dynamical peculiarities of proposed prototype are characterised by the increased energy dissipation and vibration amplitudes of the third cantilever mode as well as by the settled vibro-impacting motion at higher excitation frequencies. Thus, the research results lead to the following conclusions.

(1) Segmented electrodes of the PVEH prototype allow avoiding undesirable energy losses in the PVEH. The voltage generated by any segment of the PVEH, which features electrode segmentation at the strain nodes of the third vibration mode to improve its operation at the third resonant frequency, increased from 3.4 to 4.8 times, in comparison with the non-segmented prototype. Simultaneously, the efficiency of the energy harvester prototype also rose from 16% to 90% at lower resonant frequencies.

(2) The original experimental setup with cylindrical optics for vibro-impacting investigation of a stainless steel cantilever revealed that flexural cantilever vibrations are similar to the cantilever vibration at the third natural frequency if the support is located in the nodal point (at *x*/*l* = 0.87) of the third vibration mode. The higher vibration mode resulted in the intensification of the internal energy dissipation in the structure material, which is especially important in vibration energy harvesting.

(3) The PVEH generates higher power output values while operating at its resonant frequency. Therefore, optimal configuration of the cantilever for the defined frequency of the third (*ω*_3_ = 1335 Hz) natural transverse vibration was designed using the method of the gradient projection and a prototype was produced. The minimum cross-section of the structure that is optimal at the third natural frequency was created at the distances of 0.15*l* and 0.5*l* from the fixed end of cantilever, where *l* is its length. 

(4) Vibro-impacting laws of the optimally configured cantilever bouncing the support located at its free end were also determined experimentally. The most beneficial resonance was the third one characterised by a very large diapason of the settled vibro-impacting motion ranging from 12*ω*_1_ to 18*ω*_1_. This phenomenon could be used for increasing the efficiency of energy harvesting as well as creating high speed vibro-impacting mechanisms.

(5) Vibro-impact excitation of the cantilever-type harvester allows scavenging energy independently of the frequency of the vibrating object. This is because the third mode cantilever vibrations become prevalent after the cantilever impacts the support according to the conditions prescribed in this article.
